# Usual dietary intake and change in DNA methylation over years: EWAS in KORA FF4 and KORA fit

**DOI:** 10.3389/fnut.2023.1295078

**Published:** 2024-01-05

**Authors:** Fabian Hellbach, Dennis Freuer, Christa Meisinger, Annette Peters, Juliane Winkelmann, Ricardo Costeira, Hans Hauner, Sebastian-Edgar Baumeister, Jordana T. Bell, Melanie Waldenberger, Jakob Linseisen

**Affiliations:** ^1^Department of Epidemiology, Faculty of Medicine, University of Augsburg, University Hospital Augsburg, Augsburg, Germany; ^2^Medical Faculty, Institute for Medical Information Processing, Biometry, and Epidemiology, Ludwig-Maximilian University Munich, Munich, Germany; ^3^Institute of Epidemiology, Helmholtz Zentrum München, German Research Center for Environmental Health (GmbH), Neuherberg, Germany; ^4^Research Unit Molecular Epidemiology, Helmholtz Zentrum München, German Research Center for Environmental Health (GmbH), Neuherberg, Germany; ^5^German Center for Diabetes Research (DZD e.V.), Neuherberg, Germany; ^6^Institute of Neurogenomic, Helmholtz Zentrum München, German Research Center for Environmental Health (GmbH), Neuherberg, Germany; ^7^Technical University of Munich, Institute of Human Genetics, Klinikum Rechts der Isar, Munich, Germany; ^8^Department of Twin Research and Genetic Epidemiology, King's College London, London, United Kingdom; ^9^Else Kröner-Fresenius-Center for Nutritional Medicine, TUM School of Life Sciences, Technical University of Munich, Freising, Germany; ^10^School of Medicine, Institute of Nutritional Medicine, Technical University of Munich, Munich, Germany; ^11^Medical Faculty, Institute of Health Services Research in Dentistry, University of Münster, Münster, Germany; ^12^German Research Center for Cardiovascular Disease (DZHK), Partner Site Munich Heart Alliance, Munich, Germany

**Keywords:** DNA methylation, diet, food group, usual dietary intake, epidemiology

## Abstract

**Introduction:**

Changes in DNA methylation can increase or suppress the expression of health-relevant genes. We investigated for the first time the relationship between habitual food consumption and changes in DNA methylation.

**Methods:**

The German KORA FF4 and KORA Fit studies were used to study the change in methylation over a median follow-up of 4 years. Only subjects participating in both surveys and with available dietary and methylation data were included in the analysis (n = 465). DNA methylation was measured using the Infinium MethylationEPIC BeadChip (Illumina), resulting in 735,527 shared CpGs across both studies. Generalized estimating equation models with an interaction term of exposure and time point were used to analyze the association of 34 food groups, folic acid, and two dietary patterns with changes in DNA methylation over time.

**Results:**

The results were corrected for genomic inflation. Significant interaction terms indicate different effects between both time points. We observed only a few significant associations between food intake and change in DNA methylation, except for cream and spirit consumption. The annotated genes include CLN3, PROM1, DLEU7, TLL2, and UGT1A10.

**Discussion:**

We identified weak associations between food consumption and DNA methylation change. The differential results for cream and spirits, both consumed in low quantities, require replication in independent studies.

## 1 Introduction

DNA methylation is the most frequently researched manifestation of epigenetics because of its practical characteristics. The interindividual variation, the precision of measurement enabled by up-to-date technology, and the expected effect size of the exposure-outcome associations make DNA methylation a fitting measure for epigenetics population research (1). The methylation of cytosine–guanine sites (CpGs) is regulated by the activity of DNA methyltransferases and 10–11 translocation proteins and emerges via the transfer of the methyl group of S-adenosylmethionine (SAM). SAM is generated by the one-carbon metabolism in which a few vitamins and molecules of dietary origin act as cofactors, e.g., folic acid, vitamin B12, or methionine (2).

A change in DNA methylation can result in enhanced or suppressed gene expression. For example, cancer is often characterized by global DNA hypomethylation, while specific tumor suppressor genes are hypermethylated (3). How DNA methylation affects the corresponding gene expression is often characterized by the location (i.e., gene promoter, gene body, inside or outside of a CpG island) of the methylated CpG sites (4).

The first studies on diet and DNA methylation were conducted in cancer patients because of the known association between cancer and DNA methylation and the idea to elucidate the potentially harmful or protective effects of diet on cancer (5). Then, research started to look directly at dietary factors that influence DNA methylation and until today focused heavily on micronutrients such as folic acid or B vitamins (6). Mandaviya et al. (7) showed associations between dietary folate intake and CpG methylation in an epigenome-wide association study (EWAS), but other groups were unable to confirm these associations (8, 9). Altogether, research on DNA methylation and dietary folate intake is still inconclusive (5).

The literature provides some EWAS results for the dietary consumption of food (summarized in food groups) (9, 10). To date, no analyses have examined the longitudinal association of food consumption and temporal change of DNA methylation in mononuclear blood cells. Based on our experience in cross-sectional analyses (6, 9), we aimed to explore the interaction of diet and time on DNA methylation at the food group level by conducting EWAS on the Infinium MethylationEPIC BeadChip array with 850k CpG sites. Specifically, we assessed the association between food groups and changes in DNA methylation over time. Food groups that were analyzed in this study (i) are sources of nutrients involved in human C1 metabolism (e.g., cabbage vegetables), (ii) have a known association with systemic inflammation (e.g., red meat), or (iii) are described to be associated with metabolic disease risk (e.g., sugar-sweetened beverages). One additional aim was to test for the stability of diet and DNA methylation associations by replicating previous analyses performed in FF4 in the Fit study.

## 2 Methods

### 2.1 Population

The Cooperative Health Research in the Augsburg Region (KORA) study was conducted in the city of Augsburg and two surrounding counties in Germany. KORA FF4 is the second follow-up of the population-based health survey KORA S4 with a recruitment phase between 1999 and 2001. Initially, 4,261 randomly selected subjects aged 25–74 years agreed to participate in the S4 baseline study. In 2013/2014, 2,279 subjects participated in the second follow-up (KORA FF4). The study has been described in detail previously (11). In KORA FF4, dietary data are available for 1,602 subjects, and blood DNA samples are available for 1,928 participants, where methylation data were measured. KORA Fit is a follow-up study for all KORA study participants born between 1945 and 1964, including 707 KORA FF4 participants with dietary data and 565 with methylation data available, and a median follow-up time of 4 years. The analytic dataset included individuals who participated in KORA FF4 and KORA Fit and who had methylation and dietary intake data (*n* = 464). The investigation was conducted according to the guidelines laid down in the Declaration of Helsinki, including written informed consent of all participants. All study methods were approved by the ethics committee of the Bavarian Chamber of Physicians, Munich (EC no. 06068 and EC no. 17040).

### 2.2 Usual dietary intake

The procedure of obtaining habitual dietary intake data was identical for KORA FF4 and Fit and is described in the following. Repeated 24-h food lists and a food frequency questionnaire (FFQ) with 246 and 148 items, respectively, were used to collect dietary intake data. The 24-h food list was developed for the German NAKO Health Study (12) and collected information on food intake of the past day indicating the type of food items consumed. The FFQ was based on the German version of the multilingual European Food Propensity Questionnaire asking for the frequency of servings (13). Both were offered to the study participants as web-based forms. Two to three 24-h food lists on non-consecutive days were used to obtain the probability of consumption of food items for each subject (including the respective FFQ information as covariable). The amount consumed was estimated from the Bavarian Food Consumption Study II (BVS II), adjusting for age, sex, BMI, physical activity, and smoking status. Usual dietary intake (g/day) was estimated by multiplying the items' consumption probability with the estimated amount consumed; consumption amount times consumption probability results in continuous diet data. Modeling the usual dietary intake was done to reduce the prominent measurement error in dietary data. Supplement intake was not considered for the computation of usual dietary intake. Further information is provided elsewhere (14). Dietary data was categorized into 17 main food groups and 71 subgroups in accordance with the EPIC SOFT classification scheme (15). Nutrient data was obtained using the German Nutrient Database (Bundeslebensmittelschlüssel), version 3.02, published in May 2014 and accessed in 2019. To obtain an intake value for each food group independent of total energy intake, we used the residual method (16). Residuals are drawn from a linear regression model where usual dietary intake is the dependent variable and energy intake is the independent variable. In addition, we added the predicted food intake for the mean energy intake of the study population to the residuals for better interpretability. Two dietary patterns were used as secondary exposure variables: The Alternate Healthy Eating Index 2010 (AHEI-2010) (17) is a score used to assess consumption of foods and nutrients predictive of chronic disease risk (e.g., alcohol, grains, and fruit). A lower risk of chronic disease development is associated with a higher AHEI-2010 score. We slightly modified the AHEI-2010 since we did not have access to trans-fat consumption data and excluded it, therefore resulting in a maximum of 100 points instead of 110. For the purpose of calculating AHEI, our usual dietary intake data were transformed into servings/day with references reported in Chiuve et al. (17). The Mediterranean Diet Score (MDS) (18) depicts adherence to a dietary pattern that scores the consumption of fish, cereals, fruits and nuts, vegetables, legumes, and a high ratio of unsaturated to saturated lipids above the median intake as high (1 point) and the consumption of meat and dairy above the median as low (0 points). Thus, the MDS score reflects the individual consumption relative to the sex-specific population median of the respective food group, except for alcohol, where a moderate amount of consumption is scored highest.

### 2.3 DNA methylation data

#### 2.3.1 KORA FF4

Genomic DNA (750 ng) of 1,928 individuals was bisulfite converted using the EZ-96 DNA Methylation Kit (Zymo Research, Orange, CA, USA) in two batches (*n* = 488, *n* = 1,440). The Illumina (San Diego, CA, USA) iScan platform, in combination with the Infinium MethylationEPIC BeadChip, was used for subsequent methylation analysis according to standard protocols provided by Illumina. For generation of methylation data export files and initial quality control of assay performance, GenomeStudio software, version 2011.1, with Methylation Module, version 1.9.0, was used. Quality control and preprocessing of the data were performed in R (v3.5.1) (19) with the package minfi (v.1.28.3) [21] and primarily following the CPACOR pipeline (20). Using R commands read.metharray and bgcorrect.illumina, raw intensities were read into R and background corrected. Probes with detection *p* > 0.01 were set to missing. Before normalization, problematic samples and probes were removed, resulting in the removal of 40 samples, leaving *n* = 1,888 samples. A total of 2 samples showed a mismatch between reported sex and sex prediction by minfi; 33 had a median intensity <50% of the experiment-wide mean or <2,000 arbitrary units; and 9 (four overlapped with the previous) had >5% missing values on the autosomes. Furthermore, a total of 59,631 probes were removed (some meeting multiple criteria): probes with SNPs with minor allele frequency <5% at the CG position (*n* = 11,370), cross-reactive probes (*n* = 44,493) (21, 22), or the single base extension (*n* = 5,597) as given by minfi, and 5,786 with > 5% missing values. Ultimately, probes from the X chromosome (*n* = 17,743, following quality control) and the Y chromosome (*n* = 379) were excluded. A total of 788,106 probes remained. After dividing the signal intensities into six probe types (type II red, type II green, type I green unmethylated, type I green methylated, type I red unmethylated, and type I red methylated), quantile normalization (QN) was applied to each type (20)]. The probes of female and male participants were processed separately. The transformed intensities were then used to generate methylation beta values, a measure from zero to one indicating the percentage of cells methylated at a given locus. Beta values were controlled for outliers with ±3·*IQR* (interquartile range), resulting in the exclusion of 0.5% of the data points. The Infinium MethylationEPIC Manifest file genome build 37 (https://emea.support.illumina.com/downloads/infinium-methylationepic-v1-0-product-files.html, accessed on 14 April 2022), was used to map probes to genes. Informed consent for genetic studies was obtained from all subjects.

#### 2.3.2 KORA FIT

Quality control and preprocessing were conducted as in FF4. Before normalization, 35 samples were removed, as they had >5% missing values on the autosomes (of these, 7 also failed either the sex prediction or median intensity quality control steps implemented in minfi, commands getSex, and getQC respectively). A total of 112,150 probes were removed (some overlapping multiple categories): cross-reactive probes as given in published lists (*n* = 44,493) (20, 22); probes with SNPs with minor allele frequency >5% at the CG position (*n* = 11,370) or the single base extension (*n* = 5,597) as given by minfi; and 61,471 with >5% missing values (autosomes only). A total of 753,709 probes remained for analysis.

QN was performed as in FF4. Probes from the X chromosome (*n* = 17,743, following quality control) and the Y chromosome (*n* = 379) were excluded from the analysis.

### 2.4 Statistical analysis

We applied a generalized estimating equation (GEE) model with an exchangeable correlation matrix across all 735,527 CpG loci that were common in ~450 subjects participating in KORA FF4 and KORA Fit to examine the association between usual dietary intake (in g/day) and temporal change of methylation. The GEE model included methylation beta residual values as the dependent variable. Due to the limited power of our analysis to include the technical plate identifier variable, leading to a convergence problem, we extracted methylation residuals out of a model with methylation as the dependent variable and a plate identifier variable as the independent variable and used these values as the outcome variable. To examine the temporal change in methylation, we included an interaction term for the food exposure variables and the binary time indicator (KORA FF4/KORA Fit). The regression term for the exposure was regarded as the cross-sectional effect. The *p*-value for the interaction term was Bonferroni corrected and evaluated as significant if it was <0.05. We tested the following 34 food groups, folic acid intake, and two diet quality scores: potatoes, total vegetables, leafy vegetables, fruit vegetables, root vegetables, cruciferous vegetables, mushrooms, onions and garlic, legumes, total fruits, nuts and seeds, milk, yogurt, cheese, cream, grain products, whole grain products, total meat, fresh red meat, processed meat, total fish, eggs, plant oils, butter, margarine, total sweets, cakes, sugar-sweetened beverages, coffee, tea, wine, beer, spirits, alcohol (ethanol), AHEI, and MDS. Usual dietary intake data (residuals after regression against total energy intake) were regarded as independent variables. Covariable selection was based on the literature and our own assessment of confounding based on the disjunctive cause criterion (23). The selected covariables were sex, BMI (continuous), BMI squared, age (continuous), age squared, smoking (never, former, and current), total caloric intake (continuous), alcohol in g/day (continuous) (except for the exposures: wine, beer, spirits, AHEI, and MDS). Additionally, we measured leukocyte cell proportions in each individual (monocytes, basophil, and eosinophil granulocytes, and lymphocytes, all in percent of leukocytes) and adjusted the models with these measured leukocyte data. All variables were used at both time points. Examination of the multicollinearity of covariables led to the exclusion of neutrophil granulocytes as a covariable. The results for food groups with significant signals were corrected for genomic inflation by using the bacon package (24) in R. To test for consistency of our results, we ran a linear regression model with delta methylation value (FIT minus FF4) as the outcome and food intake in KORA FF4 as exposure, with identical FF4 covariables.

To test for the stability of cross-sectional associations obtained in a previous analysis in KORA FF4 [with a Bonferroni corrected p-value below 0.05 (6)], we applied linear regression models with these selected CpGs in KORA Fit. We applied several approaches to investigate the effect of percent change in dietary intake (Fit intake divided by FF4 intake). The KORA Fit population was analyzed as a whole and as subgroups. Subgroups were analyzed that differ in regards to their change in food consumption. The aim was to analyze subjects in isolation who had (a) stable dietary intake, i.e., <10% change in intake in the respective food group; (b) less stable dietary intake, i.e., <20% change in intake; (c) modest increase in intake, i.e., more than 10% increase in intake; (d) strong increase in intake, i.e., more than 20% increase in intake; (e) modest decrease in intake, i.e., more than 10% decrease in intake; and (f) strong decrease in intake, i.e., more than 20% decrease in intake.

We chose to correct for multiple testing by using Bonferroni correction with the number of CpG sites tested per food group as the denominator. For all analyses, only complete cases regarding the included covariates were considered. All statistical analyses were carried out with R statistical software version 4.1.2. (19).

## 3 Results

### 3.1 Longitudinal analysis

Male participants had a greater total caloric intake and alcohol intake than female participants at both time points (Table 1). More information regarding the sex- and cohort-specific consumption of all food groups is given in Supplementary Table 3. BMI was similar across both, sex and survey. On average, participants increased their physical activity from FF4 to Fit. We modeled the multiplicative interaction of food exposure and time to test the association with changes in DNA methylation. In total, we observed one significant signal each (Bonferroni corrected *p* < 0.05) for the consumption of total vegetables, fruit vegetables, root vegetables, legumes, grain products, and folic acid; three signals for mushrooms; four signals for cabbage vegetables; five signals for sugar-sweetened beverages; 122 signals for cream; and 1,630 signals for spirits (results not shown). Genes annotated to the respective CpGs were *ADAMTS2, MRC2, SECTM1, TLL2, UGT1A10, GSG1L, SLC6A13, VPS37C, COMMD5, FMN2, DLC1, PQLC3, TMEM87B*, and *LRRC34*. After correction for genomic inflation and multiple testing (Bonferroni), few results persisted with p <0.05 (Table 2): one for cabbage vegetables, fruit vegetables, grain products, sugar-sweetened beverages, and folic acid; three for cream; and 193 for spirits (Supplementary Table 1). In particular, the results with annotations of *TLL2* and *UGT1A10* survived multiple testing corrections. The linear regression analysis with delta methylation as the outcome showed that from a total of 30 significant signals from the bacon-corrected GEE analysis (only the associations with the six lowest *p*-values for cream and spirit groups), 22 were significant without correction, and 24 had the same direction in effect size (Supplementary Table 2).

**Table 1:** Demographic characteristics of all study participants with complete information needed for the GEE analysis.

	**KORA FF4**		**KORA Fit**	
	**Male**	**Female**	**Male**	**Female**
n	216	259	220	261
Age in years (median [IQR])	59.0 [54.0, 63.0]	59.0 [54.0, 63.0]	63.0 [58.0, 67.0]	63.0 [58.0, 67.0]
BMI kg/m^2^ (median [IQR])	27.4 [25.2, 30.6]	26.2 [23.3, 29.5]	27.8 [25.2, 30.8]	26.6 [23.8, 29.9]
Total calories/day (median [IQR])	2114.5 [1913.5, 2344.6]	1561.0 [1433.3, 1749.2]	2053.4 [1845.4, 2296.1]	1538.0 [1379.2, 1708.9]
Alcohol g/day (median [IQR])	14.9 [5.6, 26.0]	3.0 [1.8, 5.3]	15.0 [6.2, 25.5]	2.7 [1.6, 5.5]
**Smoking (%)**				
Regular smoker	32 (14.8)	33 (12.7)	27 (12.3)	27 (10.3)
Former smoker	107 (49.5)	91 (35.1)	113 (51.4)	96 (36.8)
Never smoker	77 (35.6)	135 (52.1)	80 (36.4)	138 (52.9)
Phys. Activ. = Active (%)	135 (62.5)	176 (68.0)	157 (71.4)	190 (72.8)
Education years = <13 years (%)	120 (55.6)	166 (64.1)	123 (55.9)	167 (64.0)

IQR, Interquartile range.

**Table 2:** GEE analysis results: significant longitudinal associations between DNA methylation sites and food group consumption.

**ProbeID**	**Food group**	**Effect size^a^**	**Standard error^a^**	***P*-value^a^**	***P-*value (bacon)^a^**	**Chr**	**RefGene name**	**RefGene group**	**Relation to CpG island**
cg25441661	Cabbage-vegetables	8.83e-04	1.50e-04	3.64e-09	1.22e-08	5	ADAMTS2^†^	Body^†^	N/A
cg01022859	Fruit-vegetables	1.52e-04	2.75e-05	2.83e-08	6.38e-08	17	MRC2	Body	N/A
cg02780269	Grain-products	3.64e-04	6.61e-05	3.75e-08	5.88e-08	17	SECTM1	TSS1500	Island
cg11613902	Cream	0.003	4.51e-04	3.51e-13	5.79e-09	10	TLL2	Body	N/A
cg11811840	Cream	−2.25e-03	3.23e-04	2.94e-12	1.86e-08	2	UGT1A10^†^	Body^†^	N/A
cg07376134	Cream	0.002	3.58e-04	1.02e-11	5.16e-08	16	GSG1L	TSS1500	Island
cg02620443	Sugar-sweetened-beverages	6.46e-05	1.07e-05	1.86e-09	1.46e-08	12	SLC6A13^†^	ExonBnd^†^	N/A
cg25479306	Spirits	0.001	1.39e-04	0.000	1.64e-15	11	VPS37C	TSS200	Island
cg24654205	Spirits	0.002	2.33e-04	0.000	2.06e-15	8	COMMD5^†^	TSS200^†^	S_Shore
cg17700903	Spirits	0.009	8.41e-04	0.000	4.55e-15	1	FMN2^†^	TSS1500^†^	N_Shore
cg08870763	Spirits	−5.72e-03	5.78e-04	0.000	1.53e-14	8	DLC1^†^	Body^†^	N/A
cg08694574	Spirits	0.008	8.04e-04	0.000	1.08e-13	16	N/A	N/A	S_Shelf
cg04731518	Spirits	−4.07e-03	4.26e-04	0.000	1.14e-13	2	PQLC3	Body	N/A
cg04439376	Spirits	0.010	0.001	0.000	1.21e-13	2	TMEM87B	TSS1500	Island
cg17737146	Spirits	0.013	0.001	0.000	1.23e-13	3	LRRC34	Body	Island
cg26942952	Folic-acid	1.76e-04	3.17e-05	3.09e-08	5.22e-08	12	N/A	N/A	S_Shore

All significant results in the GEE analysis were evaluated with the p-value for the interaction term, and all shown associations remained significant after Bonferroni and Bacon's correction. Results for consumption of spirits are shortened, due to space reasons and are accessible in Supplementary Table 2. UCSC RefGene Name—Target gene names from the UCSC database. UCSC RefGene Group—Describing CpG position. TSS1500 = 200-1500 bases upstream of the transcription start site (TSS); 5-UTR = Within the 5′ untranslated region, between the TSS and the ATG start site; Body = Between the ATG and stop codon, irrespective of the presence of introns, exons, TSS or promoters; 3′UTR = Between the stop codon and the poly A signal; ExonBnd = Exon Boundaries. Relation to UCSC CpG Island—The location of the CpG relative to the CpG Island. Shore = 0-2kb from Island; Shelf = 2–4kb from Island; N = Upstream (5′) of CpG Island; S = Downstream (3′) of CpG Island (https://knowledge.illumina.com/microarray/general/microarray-general-reference_material-list/000001568?langsel=/fo/). ^a^Values are obtained from the interaction term of the model. ^†^Indicates available splice variants.

As a means of acquiring more insight, we accessed databases such as the EWAS catalog (25), BIOS QTL browser (26), and GoDMC methylation Quantitative Trait Locus (mQTL) repository (27). The EWAS catalog showed that most CpGs were not found to be previously associated with diet-relevant or cardiometabolic phenotype outcomes. The exception was cg02620443 (sugar-sweetened beverages), which was previously associated with incident type 2 diabetes mellitus (28). Additionally, we compared our results to the significant top independent eQTM signals in the BIOS database, but none of the signals were significantly associated with local gene expression levels in the eQTMs independent top effects dataset. Six out of 16 CpGs (cg02780269 in *SECTM1*, cg11613902 in *TLL2*, cg11811840 in *UGT1A10*, cg07376134 in GSG1L, cg17737146 in *LLRC34*, and cg26942952) were found to have a genetic basis in the GoDMC database. All of the mQTLs for these six CpGs were local genetic effects (in *cis*) that annotated to the same gene as the CpG site. The finding that both genetic effects and diet may influence DNA methylation levels at these target CpG sites suggests that future work into gene-environment interactions may be advantageous in the context of identifying diet influences on the human epigenome.

### 3.2 Cross-sectional reproduction of KORA FF4 in KORA FIT

To explore the stability of associations of usual dietary intake of food groups and DNA methylation, we analyzed those food group—CpG associations in the KORA Fit study that were reported as significant in an earlier analysis of our team in KORA FF4 (6). Overall, few results could be reproduced in KORA Fit (Table 3), i.e., reached statistical significance; however, with a much smaller sample size as available in KORA FF4. Comparing the effect size estimates between KORA FF4 and KORA Fit, the size and direction were often comparable. However, the significant associations with wine intake were reproduced, and associations of beer intake and CpG DNA methylation were mostly reproduced in KORA Fit. Repeating these analyses in subgroups of KORA Fit (with an even smaller sample size) according to the change in consumption (comparing KORA FF4 and KORA Fit intake data), previous significant associations with cabbage, wine, and beer consumption were confirmed in the groups with increasing intake data. When the intake of food groups distinctly decreased in KORA Fit, the effect size estimates were no longer comparable with the FF4 results, reassuring the validity of the analytic approach (Table 3). We also evaluated the effect size of the cross-sectional term in the GEE model and observed the same direction in all but one case (not shown).

**Table 3:** Cross-sectional reproduction of significant results of food group consumption and DNA methylation in KORA FF4 in the KORA Fit study, overall, and stratified for the extent of intraindividual change in food consumption in KORA Fit when compared to KORA FF4 data.

			**Change in food group consumption (KORA Fit / KORA FF4)**								
**ProbeID**	**Food group**	**Overall**	**Stable** <±**0.1**	**Less stable** <±**0.2**	**Modest increase** ≥+**0.1**	**Strong increase** ≥+**0.2**	**Modest decrease** ≤ −**0.1**	**Strong decrease** ≤ −**0.2**	**RefGene name**	**RefGene group**	**Relation to CpG island**
cg01838728	Leafy-vegetables	−5.76e-04^#^	−1.13e-03^#^	−1.06e-03^#^	−9.83e-04^#^	−5.72e-04^#^	0.001	6.10e-04	N/A	N/A	N/A
cg15351590	Root-vegetables	−5.74e-05^#^	−2.52e-04^#^	−3.84e-04^#^	−2.04e-04^#^	−9.89e-05^#^	3.95e-04	5.08e-04	KIFC1	TSS1500	N_Shore
cg14698575	Cabbage-vegetables	6.01e-04^#^	−8.45e-04	6.78e-04^#^	0.001^*#^	0.001^*#^	−2.02e-04	−3.19e-04	N/A	N/A	S_Shore
cg23709902	Cabbage-vegetables	2.35e-04^#^	0.001^#^	0.002^*#^	1.85e-04^#^	2.86e-04^#^	7.44e-04^#^	7.95e-04^#^	SRCIN1	Body	Island
cg06102690	Cabbage-vegetables	−3.57e-04	−6.50e-05	2.28e-04^#^	−6.32e-04	−2.58e-04	5.47e-04^#^	3.87e-04^#^	CCDC149	TSS200	N/A
cg10399824	Onions-garlic	−2.10e-04^#^	−2.65e-04^#^	−4.90e-04^#^	−7.60e-05^#^	−1.36e-04^#^	9.49e-05	1.26e-04	GRK5	Body	N/A
cg06690548	Wine	−1.74e-04^*#^	−9.61e-05^#^	−1.52e-04^#^	−2.12e-04^*#^	−2.15e-04^*#^	−2.58e-04^*#^	−2.42e-04^*#^	SLC7A11	Body	N/A
cg06690548	Beer	−5.02e-05^*#^	−4.60e-05^*#^	−5.41e-05^*#^	−3.59e-05^#^	−3.19e-05^#^	−5.11e-05^*#^	−5.25e-05^#^	SLC7A11	Body	N/A
cg26457483	Beer	−1.92e-05^#^	−5.01e-05^#^	−4.68e-05^*#^	−1.11e-06^#^	−4.61e-06^#^	4.48e-05	1.25e-04^*^	PHGDH	Body	S_Shore
cg14476101	Beer	−2.38e-05^#^	−5.21e-05^#^	−4.65e-05^*#^	−6.90e-07^#^	3.73e-06	1.51e-05	7.87e-05	PHGDH	Body	S_Shore
cg06088069	Beer	−3.69e-05^*#^	−5.03e-05^*#^	−4.42e-05^*#^	−2.80e-05^*#^	−1.62e-05^#^	−8.21e-06^#^	−2.08e-06^#^	JDP2^†^	5′UTR^†^	S_Shore
cg16246545	Beer	−7.05e-06^#^	−4.27e-06^#^	−1.14e-05^#^	2.00e-07	−2.54e-06^#^	1.90e-05	7.66e-05^*^	PHGDH	Body	S_Shore
cg15837522	Beer	−8.18e-05^*#^	−4.93e-05^#^	−5.67e-05^#^	−9.78e-05^*#^	−1.01e-04^*#^	−6.03e-05^#^	1.20e-05	N/A	N/A	N/A
cg18120259	Beer	−3.22e-05^*#^	−4.82e-05^#^	−6.05e-05^*#^	−1.26e-05^#^	−4.03e-06^#^	−4.32e-05^#^	1.85e-05	LOC100132354	Body	N/A
cg08228578	Beer	−2.38e-05^*#^	−2.46e-05^#^	−2.06e-05^#^	−2.48e-05^#^	−2.54e-05^#^	−1.43e-06^#^	−2.00e-05^#^	SHMT2^†^	Body^†^	S_Shore
cg10223198	Beer	−2.09e-05^*#^	−5.13e-05^*#^	−2.02e-05^#^	−5.38e-06^#^	−1.43e-05^#^	−4.29e-07^#^	−1.58e-05^#^	N/A	N/A	N/A

Effect sizes of the linear regression models adjusted for possible confounders at time point KORA Fit. We applied several approaches to investigate the effect of percent change in dietary intake (Fit intake divided by FF4 intake). The aim was to analyze subjects in isolation who had (a) stable dietary intake, i.e., <10% change in intake in the respective food group; (b) less stable dietary intake, i.e., <20% change in intake; (c) modest increase in intake, i.e., more than 10% increase in intake; (d) strong increase in intake, i.e., more than 20% increase in intake; (e) modest decrease in intake, i.e., more than 10% decrease in intake; (f) strong decrease in intake, i.e., more than 20% decrease in intake. UCSC RefGene Name—Target gene names from the UCSC database. UCSC RefGene Group—Describing CpG position. TSS1500 = 200–1500 bases upstream of the Transcription start site (TSS); 5-UTR = Within the 5′ untranslated region, between the TSS and the ATG start site; Body = Between the ATG and stop codon, irrespective of the presence of introns, exons, TSS or promoters; 3′UTR = Between the stop codon and the poly A signal; ExonBnd = Exon Boundaries. Relation to UCSC CpG Island—The location of the CpG relative to the CpG Island. Shore = 0–2kb from Island; Shelf = 2–4kb from Island; N = Upstream (5′) of CpG Island; S = Downstream (3′) of CpG Island (https://knowledge.illumina.com/microarray/general/microarray-general-reference_material-list/000001568?langsel=/fo/). ^*^Indicates significance in the Fit cohort without correction. ^#^Indicates the same direction of effect sizes in the analysis in the FF4 and Fit cohort. ^†^indicates available splice variant.

## 4 Discussion

This is the first EWAS examining associations of a broad range of food groups and changes in DNA methylation over time. Our results indicate that there are differences in effect size for some diet-DNA methylation associations dependent on the time point, which also implies that most other associations do not differ in effect size, although this cannot be said with statistical certainty. We observed very few signals for the consumption of various food groups and many signals for cream and spirit consumption. Our analysis of (cross-sectional) stability of dietary exposure and DNA methylation associations showed that most signals were not stable over time, although this needs to be taken with caution due to the limited power of the analysis.

The by far highest number of significant longitudinal associations were observed for spirit consumption. This could be due to the alcohol content, which is known for associations with DNA methylation (29), but this point does not hold for the other alcoholic beverages that were examined (i.e., wine, beer, ethanol in g/day). The usually small amounts of spirits consumption (median ± IQR; FF4: 0.3 g/day ±0.2; FIT: 0.3 g/day ±0.2, Supplementary Table 1) and the fact that the vast majority of signals disappeared after correction for genomic inflation (Supplementary Table 2) indicates that these results could partially be false positives. However, a few signals remained significant and are discussed in the following. The cg20997359-associated gene *MAP1B* was found to be associated with global developmental delays, seizures, and dysmorphic features due to a *MAP1B* non-sense mutation in a case study (30). The gene *CLN3* annotated to one of the CpGs showed a significant association with alcohol exposure in one GWAS as indicated in the GWAS catalog (31). Cg21230392 is mapped to the *PROM1* gene, which is a relevant progenitor marker in human alcoholic hepatitis (32) and showed increased expression in mice fed with alcohol (33). The longitudinal association indicates increased methylation at the time of the Fit survey which some studies show can result in gene suppression due to the CpG positioned in the first exon (34). Another CpG was associated with the *DLEU7* gene, which was shown to be associated with snoring (35) and therefore this epigenetic mechanism could be related to alcohol-induced snoring (36).

Habitual cream consumption was low (median ± IQR; FF4: 1.4 g/day ± 1.15; FIT: 1.3 g/day ± 0.8), indicating a low heterogeneity between subjects, which, in turn, may increase the risk of misclassification and thus less reliable results. Cream is closely related to milk which may modify human serum cholesterol levels. The gene *TLL2* (cg11613902) was observed to be significantly positively associated in an African GWAS with total cholesterol as the exposure (37). We observed decreasing methylation with increasing cream consumption in FF4. This gene alters a regulatory motif for sterol regulatory element-binding proteins (38), which are crucial for lipid metabolism. Another observation was the association of cg11811840 methylation and cream consumption. The CpG is mapped to *UGT1A10* and is involved in xenobiotic metabolism. It was found that *UGT1A* locus activity is related to fibrosis in nonalcoholic steatohepatitis, where higher activity induced by a SNP allele leads to protection from this disease (39). The interaction effect suggests higher *UGT1A10* activity for the FIT survey due to lower methylation levels in the gene body.

The CpG with the lowest *p*-value for the association of DNA methylation and sugar-sweetened beverage consumption maps to the gene *SLC6A13*, which is an amino acid transmembrane transporter and monocarboxylic acid transmembrane transporter. *ADAMTS2* is the gene that is annotated to the top signal (cg25441661) in the analysis of cabbage vegetables and DNA methylation, which translates to a metalloproteinase with thrombospondin motifs. Minor zinc levels in cabbage could be one factor for this identified association because of the zinc-cofactor binding site found in the *ADAMTS2* protein (40). The CpG which showed an association with folic acid does not map to any known gene. Mapped genes to the association of fruit vegetables and grain products are *MRC2* and *SECTM1* and are members of the mannose receptor family of proteins and transmembrane and secreted protein with characteristics of type 1a transmembrane protein.

Although the association of DNA methylation at cg26577993 (mapped gene: *TUBB3*) and legume consumption did not survive genomic inflation correction, it is worth mentioning that one study found that *TUBB3* expression was modified by the application of lupeol, found in the skin of lupin seeds, to rat cerebral cultures submitted to inflammatory damage (41). Additionally, another study found *TUBB3* expression changes in rat cortical astrocyte/neuron primary co-cultures after the application of monocrotaline, which is a toxic substance in a plant from the family of Leguminosae (42). Our results showed only a few stable dietary DNA methylation associations between the FF4 and Fit surveys. This could be due to the limited sample size of the KORA Fit study. Past EWAS in FF4 (6) on its own and a meta-analysis including the FF4 cohort (9) results have shown different signals for different food groups.

Several strengths accompany this study. We filtered robust findings by applying bacon correction to address genomic inflation by estimating the empirical null distribution. Many signals lost statistical significance, but a few persisted. Additionally, GEE models are robust to the misspecification of the correlation structure. Finally, we used habitual dietary intake data to represent dietary information. This approach decreases the prominent measurement bias in dietary questionnaire data by using a blended approach combining multiple information sources (i.e., 24-h recalls and FFQs).

Our study is also subject to certain limitations. The longitudinal study design of KORA FF4 and Fit is inherent to two biases. Recall bias could lead to less accurate reporting of dietary or lifestyle information in the follow-up, and selection bias could stem from the fact that health-conscious people tend to commit more frequently to follow-up examinations. Additionally, we have no gene expression data available, and the data stems only from blood cells, so tissue-wide extrapolation of the results is not possible. Furthermore, the sample size of subjects in KORA FF4 and Fit combined is small, and therefore, replication is needed.

## 5 Conclusion

This study aimed to investigate the longitudinal association of usual dietary intake and DNA methylation in blood mononuclear cells. We observed several significantly different marginal effect sizes in the GEE model, especially with spirits and cream consumption, of which several persisted after correction for genomic inflation. As spirits and cream are consumed in low amounts, we cannot exclude spurious findings. Our earlier reported cross-sectional findings on diet-DNA methylation associations were largely not statistically significant (but similar in size and direction of the effect size estimate) in KORA Fit, which is likely due to the small sample size. With decreasing dietary intake (from FF4 to Fit) of food groups, effect size estimates seem to change direction. Finally, it is important that our longitudinal results are reproduced in larger cohorts to ensure sufficient statistical power.

## Data availability statement

The data analyzed in this study is subject to the following licenses/restrictions: the informed consent given by KORA study participants does not cover data posting in public databases. However, data are available on request from KORA-gen (https://helmholtz-muenchen.managed-otrs.com/external/, accessed on 03.05.2023). Data requests can be submitted online and are subject to approval by the KORA Board. Requests to access these datasets should be directed to https://helmholtz-muenchen.managed-otrs.com/external.

## Ethics statement

The studies involving humans were approved by the Ethics committee of the Bavarian Chamber of Physicians, Munich. The studies were conducted in accordance with the local legislation and institutional requirements. The participants provided their written informed consent to participate in this study.

## Author contributions

FH: Conceptualization, Formal analysis, Investigation, Methodology, Writing—original draft, Writing—review & editing. DF: Methodology, Writing—review & editing. CM: Investigation, Methodology, Writing—review & editing. AP: Data curation, Writing—review & editing. JW: Data curation, Writing—review & editing. RC: Methodology, Writing—review & editing. HH: Supervision, Writing—review & editing. S-EB: Methodology, Supervision, Writing—review & editing. JB: Methodology, Writing—review & editing. MW: Data curation, Methodology, Writing—review & editing. JL: Conceptualization, Data curation, Funding acquisition, Supervision, Writing—review & editing.
